# Supplementary material for: miRNAs, target genes expression and morphological analysis on the heart in gestational protein-restricted offspring
Source: PLoS One. 2019 Apr 29;14(4):e0210454. doi: 10.1371/journal.pone.0210454 (PMC6507319; doi:10.1371/journal.pone.0210454)
Supplement: S1 Fig — (A) MiRNAs volcano plot in LP-12d versus LP-12d groups; (B) MiRNAs volcano plot in LP-16w versus LP-16w groups. Balls above the red dashed line indicates the miRNAs differentially expressed between groups. (DOCX) [file pone.0210454.s001.docx]

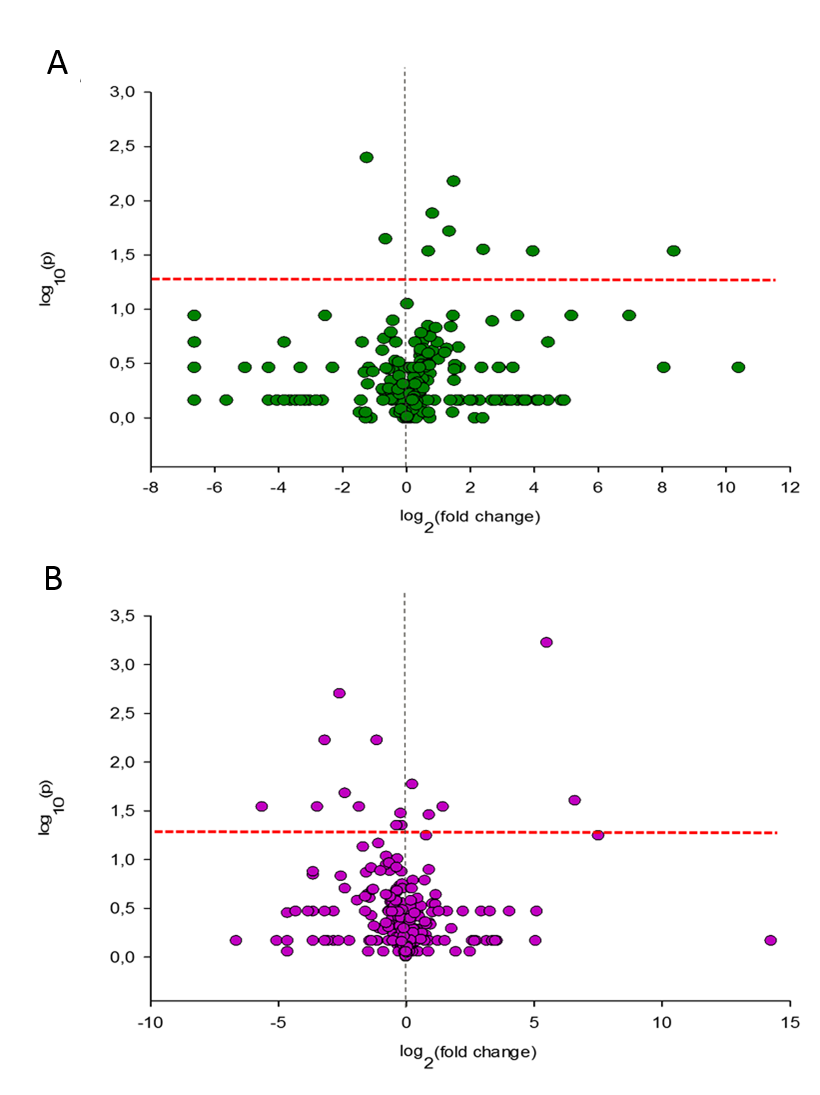


**S1 Fig. Volcano plot analysis of 12 days and 16 weeks old animals.**

(A) LP-12d versus NP-12d miRNA fold-change volcano plot; (B) LP-16w versus NP-16w miRNA fold-change volcano plot. Balls above the red dashed line indicates the miRNAs differentially expressed between groups.
