# Supplementary material for: miRNAs, target genes expression and morphological analysis on the heart in gestational protein-restricted offspring
Source: PLoS One. 2019 Apr 29;14(4):e0210454. doi: 10.1371/journal.pone.0210454 (PMC6507319; doi:10.1371/journal.pone.0210454)
Supplement: S2 Fig — (A) BBS1 blots of 12 days old offspring; (B) BBS1 blots of 16 weeks old offspring; (C) Calml3 blots of 12 days old offspring; (D) Calml3 blots of 16 weeks old offspring; (E) Dnmt3a blots of 12 days old offspring; (F) Dnmt3a blots of 16 weeks old offspring; (G) Oxct1 blots of 12 days old offspring; (H) Oxct1 blots of 16 weeks old offspring; (I) Rictor blots of 12 days old offspring; (J) Rictor blots of 16 weeks old offspring. (DOCX) [file pone.0210454.s002.docx]

**
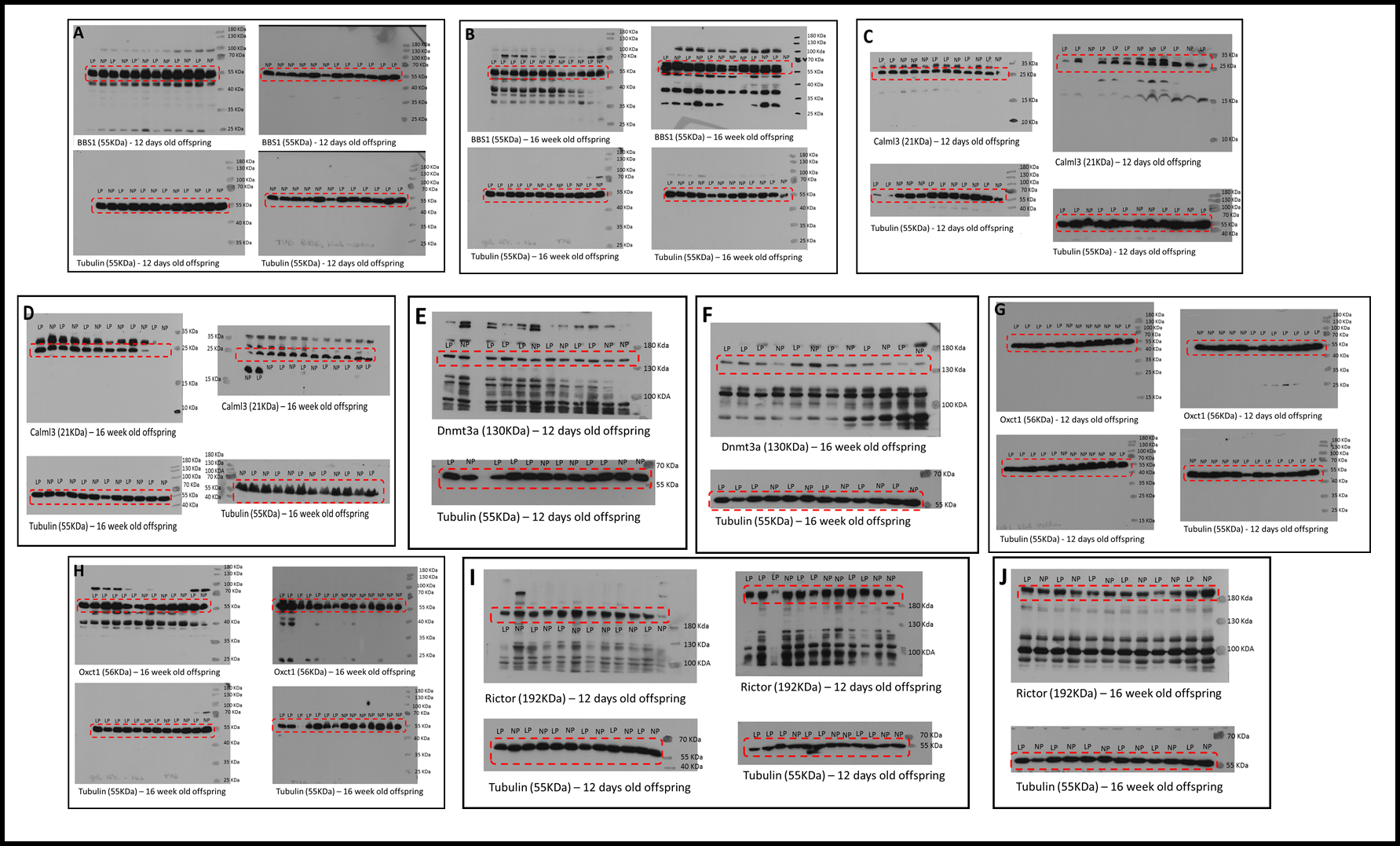
**

**S2 Fig. Western blot gels.**

(A) BBS1 blots of 12 days old offspring; (B) BBS1 blots of 16 weeks old offspring; (C) Calml3 blots of 12 days old offspring; (D) Calml3 blots of 16 weeks old offspring; (E) Dnmt3a blots of 12 days old offspring; (F) Dnmt3a blots of 16 weeks old offspring; (G) Oxct1 blots of 12 days old offspring; (H) Oxct1 blots of 16 weeks old offspring; (I) Rictor blots of 12 days old offspring; (J) Rictor blots of 16 weeks old offspring.
