# Supplementary material for: miRNAs, target genes expression and morphological analysis on the heart in gestational protein-restricted offspring
Source: PLoS One. 2019 Apr 29;14(4):e0210454. doi: 10.1371/journal.pone.0210454 (PMC6507319; doi:10.1371/journal.pone.0210454)
Supplement: S1 Table — (DOCX) [file pone.0210454.s003.docx]

| **S1 Table.** **Primers sequences** | | |
| --- | --- | --- |
| **Gene Symbol** | **Forward Primer** | **Reverse Primer** |
| **Adrbk1** | GCCTAGTCTCCGATTGACATC | GATTGACCGCATGACATTGAC |
| **Akap12** | GGCTCTGAAATCTTGGTCTCA | GACCTGGCAGAATCCTAAGATG |
| **Amotl1** | AGTGGTTGCTGATGGTAGTG | CAAATGGGAGCAGAAGTACCT |
| **Bbs1** | CCTTGTTCTCAGTGCCTAGC | TGAACCAGCACAAGTCCAAG |
| **Calml3** | CTACTCCACCAGGGTTCATTTC | GTGTGTGGGAAGAAGAGAGAAG |
| **Dab2** | AGATCCTTTGCTTGTGCTGT | GTATTGATGATGTGCCTGATGC |
| **Dnmt3a** | CCACATGAGATACAAATGTCCTC | CAGAAAGAGCACAACGAGAAAC |
| **Gapdh** | GTAACCAGGCGTCCGATAC | TCTCTGCTCCTCCCTGTTC |
| **Gpr22** | TGGTATCGATGTCCTCAATGTC | CCATAAAGCAACAGCGAACAG |
| **Hbegf** | GACACCTCTGTCCATGGTAAC | GAAAGAAGAGAGATCCATGCCT |
| **Hic2** | CTGTTTTGAATGGCTGGGAAG | AGGAAGTCTGTGTGCTCAAG |
| **Inppl1** | CCCACAGCTCCCTTGTTC | GGATTACCGTCCGATTGCT |
| **Insr** | GCCATCAGTTCCATCACTACC | TCAATGAGTCAGCCAGTCTTC |
| **Jcad** | GAACCGAGAGGAAGTCAAGAAC | CCCACCATTATCCACCCTTATG |
| **Mcf2l** | ATGACCAAGATGAACCCGATG | CACCTTCCCAGACTACCCA |
| **Mmp8** | TGGCAGCATCAAATCTCAGG | CAGCACCTATTCACTACCTCAA |
| **Nfat5** | ACACTTCTTCCTCTCCTTTCAC | ATGGCTCTACTCTGACATTGC |
| **Odc1** | CGAGGATATGGCAGTCAAACTC | AGGAGACAGCATTCAGAGTTG |
| **Oxct1** | CCTGTGCTCTTCCTGATGTC | CGTCAACCGCATCATTACAGA |
| **Ppp2ca** | CCACTGATACAATTCAAACCTGAC | CAGAACTGTCTCCCTCCTGTA |
| **Rictor** | AACCGTGCTTCTCTGTCTTC | AGTATGTGCGAGCTGATGTAG |
| **Sirt1** | AATGTAGATGAGGCAGAGGTC | TCTGAAAGTAAGACCAGTAGCAC |
| **Tgfbr1** | GAAGGTACAAGATCATAGTAAGGCA | CAAACGTGCTGACATCTATGC |
| **Trps1** | CCGCCATTTGCATTCTTTCG | GGCAAGACCAAGGACGAATC |
| **Ubn1** | GACTTATAGGATGAAGACAGCAGAC | GCAGTTAGTAGTGTGACATCGT |
