# Supplementary material for: miRNAs, target genes expression and morphological analysis on the heart in gestational protein-restricted offspring
Source: PLoS One. 2019 Apr 29;14(4):e0210454. doi: 10.1371/journal.pone.0210454 (PMC6507319; doi:10.1371/journal.pone.0210454)
Supplement: S2 Table — (DOCX) [file pone.0210454.s004.docx]

| **S2 Table**. Targets mRNA expression values of NP-12d, LP-12d, NP-16w and LP-16w groups. | | | | | | |
| --- | --- | --- | --- | --- | --- | --- |
|  | **NP-12d** | **LP-12d** | **p-value** | **NP-16w** | **LP-16w** | **p-value** |
| **Adrbk1** | 1.00±0.23 | 0.99±0.32 | 0.937 | 1.00±0.23 | 1.28±0.21 | **0.038** |
| **Akap12** | 1.00±0.24 | 1.10±0.40 | 0.570 | 1.00±0.51 | 0.73±0.13 | 0.227 |
| **Amotl1** | 1.00±0.27 | 1.05±0.44 | 0.803 | 1.00±0.25 | 1.07±0.28 | 0.647 |
| **Bbs1** | 1.00±0.38 | 0.66±0.20 | **0.04** | 1.00±0.20 | 1.50±0.33 | **0.004** |
| **Calml3** | 1.00±0.36 | 0.51±0.16 | **0.01** | 1.00±0.66 | 0.90±0.58 | 0.802 |
| **Dab2** | 1.02[0.91-1.09] | 0.93[0.80-1.21] | 0.867 | 0.91[0.67-1.23] | 1.06[0.90-1.39] | 0.240 |
| **Dnmt3a** | 1.00±0.52 | 2.30±0.70 | **0.001** | 1.00±0.44 | 1.54±0.49 | **0.049** |
| **Gpr22** | 0.83[0.59-1.67] | 0.56[0.43-0.96] | 0.138 | 1.00±0.48 | 2.12±0.86 | **0.019** |
| **Hbegf** | 0.98[0.85-1.19] | 1.02[0.75-1.57] | 0.805 | 1.00±0.51 | 1.50±0.58 | 0.116 |
| **Hic2** | 1.00±0.36 | 1.05±0.46 | 0.809 | 1.00±0.56 | 0.69±0.30 | 0.225 |
| **Inppl1** | 0.97[0.92-1.13] | 0.92[0.87-1.08] | 0.463 | 1.00±0.31 | 1.46±0.22 | **0.015** |
| **Insr** | 1.00±0.22 | 0.91±0.19 | 0.453 | 1.00±0.23 | 1.04±0.23 | 0.760 |
| **Jcad** | 1.02[0.89-1.08] | 0.95[0.76-1.20] | 0.662 | 1.00±0.34 | 1.00±0.32 | 0.982 |
| **Mcf2l** | 1.00±0.31 | 0.85±0.36 | 0.411 | 1.00±0.25 | 0.90±0.26 | 0.530 |
| **Mmp8** | 1.07[0.60-1.35] | 0.68[0.51-1.91] | 1.000 | 1.00±0.52 | 1.44±0.62 | 0.244 |
| **Nfat5** | 1.09[0.75-1.25] | 1.36[0.73-1.48] | 0.281 | 1.00±0.45 | 0.84±0.50 | 0.524 |
| **Odc1** | 0.96[0.81-1.24] | 1.06[0.34-1.61] | 1.000 | 0.92[0.88-1.11] | 1.39[0.96-2.07] | 0.138 |
| **Oxct1** | 1.00±0.21 | 1.49±0.33 | **0.011** | 1.00±0.19 | 1.32±0.20 | **0.005** |
| **Ppp2ca** | 1.00±0.30 | 1.34±0.54 | 0.173 | 1.00±0.23 | 1.25±0.40 | 0.144 |
| **Rictor** | 1.00±0.16 | 1.43±0.45 | **0.028** | 1.00±0.49 | 1.45±0.70 | 0.190 |
| **Sirt1** | 1.00±0.17 | 1.22±0.48 | 0.255 | 1.00±0.20 | 0.99±0.24 | 0.956 |
| **Tgfbr1** | 1.00±0.35 | 0.98±0.33 | 0.906 | 0.94[0.69-1.26] | 0.71[0.55-1.53] | 0.535 |
| **Trps1** | 1.00±0.39 | 1.56±0.44 | **0.021** | 0.94[0.61-1.65] | 0.71[0.60-1.53] | 0.902 |
| **Ubn1** | 1.00±0.37 | 1.31±0.38 | 0.126 | 1.00±0.24 | 0.84±0.34 | 0.349 |
| Data are expressed as the mean ± SD or as the median [lower quartile - upper quartile]. | | | | | | |
